# Effect of Screw Distribution on Stability and Interfragmentary Strain of Lower Tibial Fractures: A Finite Element Analysis

**DOI:** 10.1007/s11596-025-00116-1

**Published:** 2025-09-19

**Authors:** Huan Su, Huan Xiao, Jian-jun Zhou, Fang Lei, Liang Liang, De-wei Wang

**Affiliations:** 1 Department of Bone and Joint Surgery, Bijie Traditional Chinese Medicine Hospital, Bijie, 551700 China; 2 Second Department of Orthopedics, Fifth Affiliated Hospital of Zunyi Medical University, Zhuhai, 519100 China; 3 Department of Orthopedics, Hengqin Hospital, The First Affiliated Hospital of Guangzhou Medical University, Zhuhai, 519000 China

**Keywords:** Locking compression plate, Working length, Lower tibial fractures, Distal tibial fractures, External fixator, Finite element analysis, Biomechanics, Interfragmentary strain, Screw distribution, Screw configuration, Fracture healing

## Abstract

**Objective:**

The aim of this study was to explore the influence of working length (determined by the screw position) on the stiffness and interfragmentary strain (IFS) of femoral locking compression plate (LCP) external fixators for lower tibial fractures under full weight-bearing conditions, with the goal of providing a reference basis for clinical applications.

**Methods:**

Finite element analysis software was used to construct a model of a lower tibial fracture with external femoral LCP fixation. The models were divided into four groups according to the different working lengths (external femoral locking plate fixation 1 [EF1], EF2, EF3, and EF4). Stress distribution clouds, fracture end displacements, stiffness and IFS were tested for each model group at different loads.

**Results:**

Compared with those in the EF1 group, the stiffnesses in the EF2, EF3, and EF4 groups decreased by 28%, 31%, and 37%, respectively, under axial compression loading. Compared with those in the EF1 group, the stiffnesses in the EF2, EF3, and EF4 groups decreased by 19%, 33%, and 35%, respectively, under axial torsion loading. Compared with those in the EF1 group, the stiffnesses in the EF2, EF3, and EF4 groups decreased by 32%, 33%, and 35%, respectively, under a three-point bending load. The IFS of the four finite element models increased with the working length of the plate, with EF1 (76%) < EF2 (107%) < EF3 (110%) < EF4 (122%). Finite element analysis revealed that under full weight-bearing conditions, the structural stiffness of the femoral LCP external fixator decreased with increasing working length, leading to an increase in the IFS, which resulted in an IFS that exceeded the ideal range required for secondary healing.

**Conclusion:**

For unstable lower tibial fractures, screws in the femoral LCP external fixator should be placed as close to the fracture end as possible to increase stability and promote fracture healing.

## Introduction

Fractures of the distal tibia account for 3%–10% of all tibial fractures, whereas open tibial fractures make up approximately 30% of fractures [1, 2]. The tibia is located superficially and has a thin, soft tissue covering, making patients susceptible to open fractures and severe soft tissue injuries caused by high-energy trauma. The main treatment methods are debridement and external fixation [2–7]. External fixation is a minimally invasive method that causes minimal damage to the surrounding soft tissues and preserves the blood supply near the fracture site. It is a simple, quick, and easily adjustable treatment option [3, 8–10]. Therefore, it is often used as a temporary or ultimate treatment for open fractures or closed fractures with poor soft tissue conditions. However, traditional external fixation devices are bulky and large, which can be inconvenient for patients. When used to treat lower tibial fractures, cross-joint fixation is often necessary. However, prolonged fixation can lead to irreversible joint stiffness and functional impairments [3, 5, 8–10].

The locking compression plate (LCP) offers the advantages of a low profile and angular stability. The principle of LCP external fixation is the same as that of an external fixation frame, and its stability mainly depends on the screw holding power [5, 9]. Therefore, to reduce complications, several surgeons have attempted to use distal femoral LCP external fixation for distal tibia fractures in certain patients and have achieved satisfactory clinical results [9–19]. However, only a few case reports and simple biomechanical studies of the distal femoral LCP exist. Therefore, there is not enough biomechanical evidence to prove that the external fixation technique for the femoral LCP is safe and effective [18–24]. Previous studies have investigated the effects of fracture gap size and plate spacing on the structural stability of femoral LCP external fixation for lower-end tibia fractures [18–24]. However, there has been no study on the effect of working length (the distance between the nearest screws and either side of the fracture end) on the structural stability of femoral LCP external fixation for the treatment of fractures of the lower end of the tibia. Therefore, in this study, we propose for the first time to model tibial fractures under femoral LCP external fixation using the finite element method to investigate the effect of plate working length on structural stability. The results provide a biomechanical basis for treating lower tibia fractures.

## Materials and Methods

### Three-Dimensional Modeling

In this study, a 2D computed tomography (CT) image dataset was obtained by scanning the right lower limb of a normal male. A three-dimensional (3D) geometric model of the tibia was then reconstructed from the CT images using the 3D model reconstruction software Mimics (software version 19.0; Materials, Belgium), which included the contours of the cortical and cancellous bone. The 3D geometric model of the tibia was subsequently optimized using Geomagic Studio software (software version 2012, USA) for noise reduction, encapsulation, smooth restoration, and surface fitting to construct a model of the right tibia with a geometric shape that highly conformed to the physical specimen. The tibia model was imported into the computer-aided design (CAD) software Creo 5.0 (software version 5.0; PTC, Inc., USA), and a transverse fracture with a 10 mm defect was generated at a distance of 50 mm from the lower tibial metaphysis (50 mm above the ankle joint) to simulate an unstable fracture model of the lower tibia [19, 24]. On the basis of the anatomical parameters of the left distal femoral LCP (Johnson & Johnson, 18 holes, distal 7 proximal 11) provided by the manufacturer and self-measured dimensions, the LCP locking system was modeled at a 1:1 ratio using Creo 5.0 software. The locking screw geometry was simplified to a cylinder with a length of 50 – 60 mm (diameter = 5 mm) to reduce the calculation time.

### Finite Element Model

**Fig. 1:**
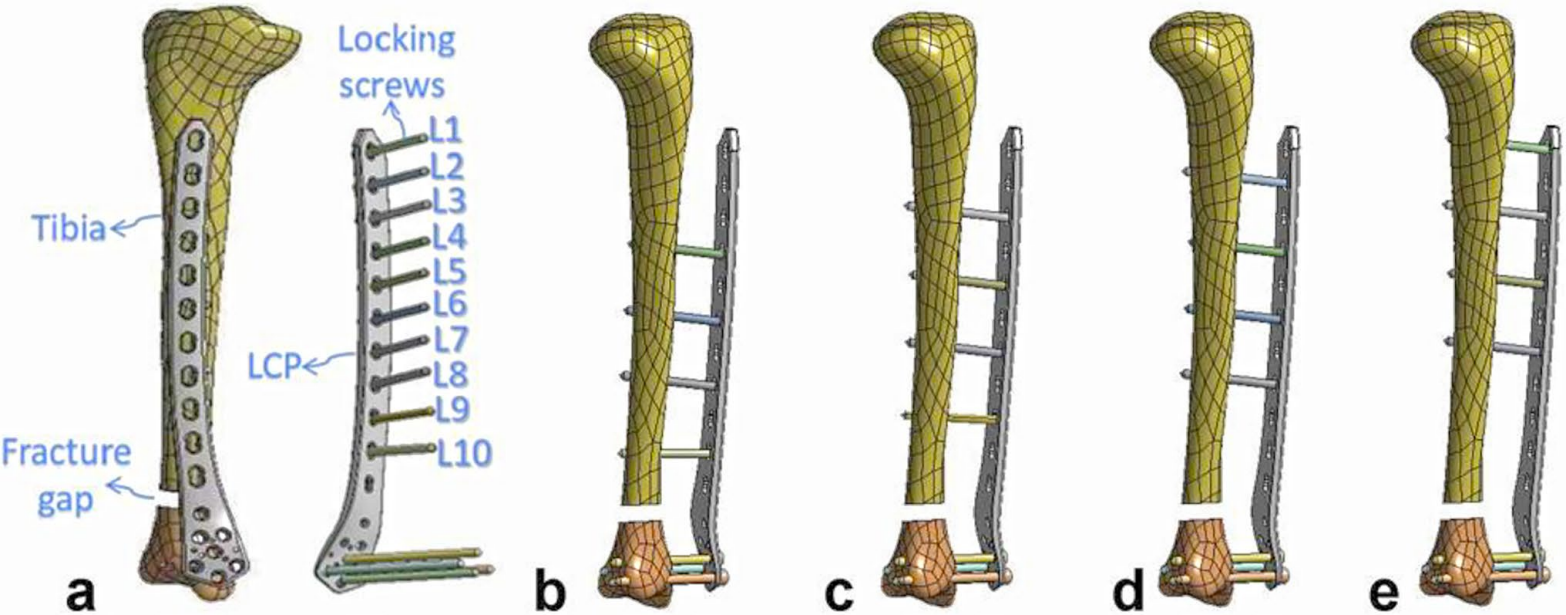
Finite element model of each group assembly. **a** Finite element model of lower tibial fractures and the femoral LCP locking system; **b** EF1 group 4–6-8–10; **c** EF2 group 3–5-7–9; **d** EF3 group 2–4-6–8; **e** EF4 group 1–3-5–7

With Creo 5.0 software, the femoral LCP was fixed 30 mm from the tibial bone surface, and four locking screws were used to fix the two ends of the fracture in each model [19, 22, 24]. Four different working lengths of the plate construct were defined (group external femoral locking plate fixation 1 [EF1, 6 cm], group EF2 [8 cm], group EF3 [10 cm], and group EF4 [12 cm]). Throughout the entire analysis process, the position of the distal screws remained fixed. The screw holes at the proximal end of the femoral LCP were labeled L1–L10 (Fig. 1a), and the proximal screws in the four groups of models were fixed at distances of 1, 2, 3, and 4 holes from the fracture end (proximal screw distributions: EF1 group 4–6-8–10, EF2 group 3–5-7–9, EF3 group 2–4-6–8, and EF4 group 1–3-5–7) (Fig. 1b–1e).

The above 4 sets of finite element models were imported into the software ANSYS 15.0 Workbench (software version 15.0, ANSYS Company, USA) for processing. Binding between the contact interfaces, between the screw and LCP, and between the screw and bone was established according to the boundary conditions defined in previous studies [5, 22]. In this study, all the materials were considered homogeneous, continuous, and isotropic linear elastic elements. The specific material properties are shown in Table 1. The mesh module of ANSYS DS software was subsequently used to divide each experimental model into 10-node quadratic tetrahedral meshes. The numbers of nodes and elements obtained after mesh division are shown in Table 2.

**Table 1:** Material properties

Material name	Cortical bone	Cancellous bone	Fixation
Modulus of elasticity	17 GPa	1.1 GPa	110 GPa
Poisson's ratio	0.3	0.3	0.3

**Table 2:** Number of nodes and meshes

Items	Tibia	Fixation
Knots	1,056,566	254,998
Units	730,084	162,998

### Boundary and Loading Conditions

**Fig. 2:**
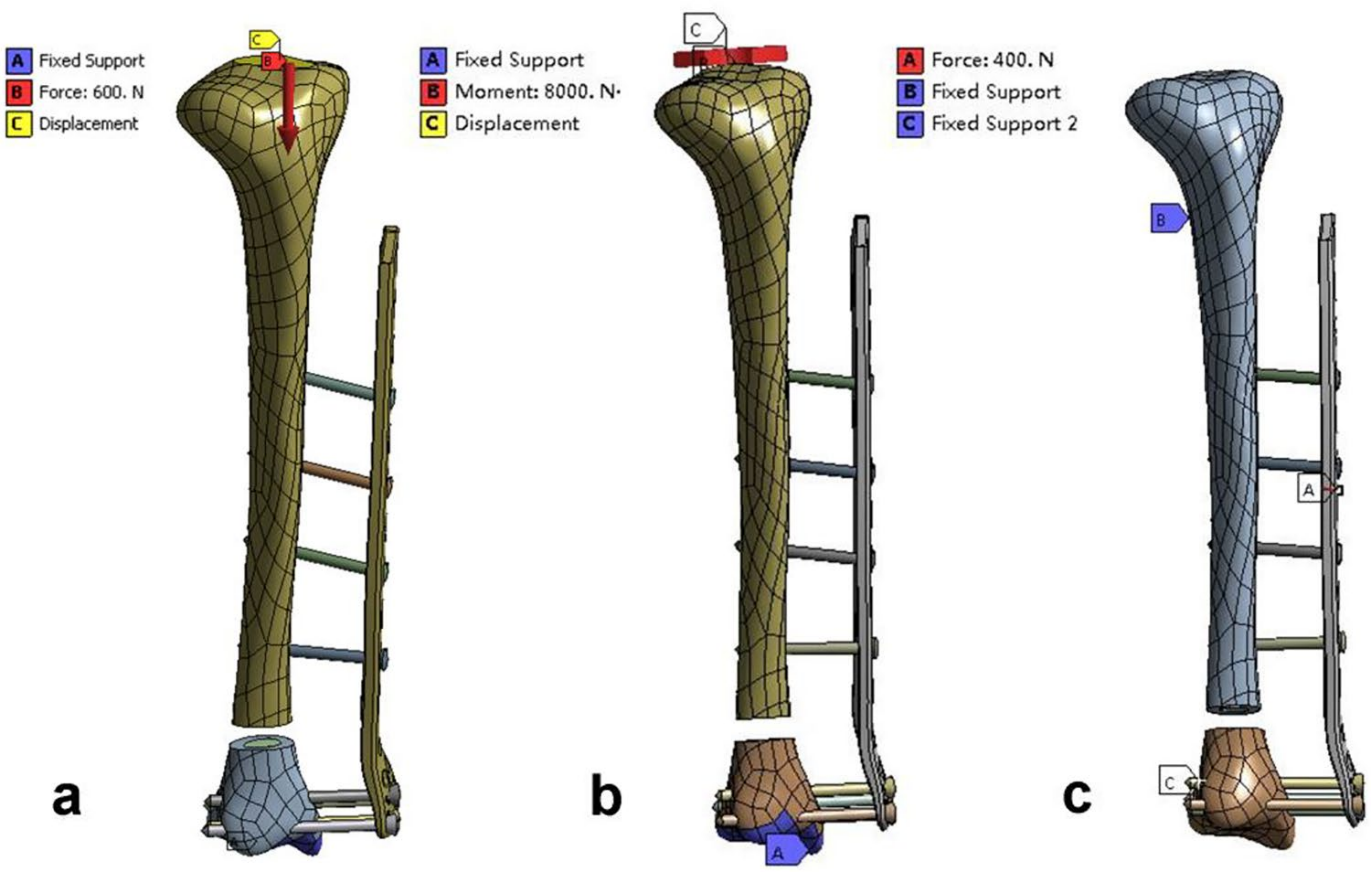
Boundary and loading conditions. **a** compression; **b** torsion; and **c** three-point bending

Axial Compression Load: The lower tibia was set with fixed constraints, and an axial load of 600 N was applied to the tibial plateau to simulate weight-bearing when standing, which was equivalent to one time the body weight (Fig. 2a).

Axial Torsion Load: The lower tibia was set with fixed constraints, and an external or internal rotation force of 8 N•M was applied to the tibia to simulate the axial torsion force at the knee joint during adult activities (Fig. 2b).

Three-Point Bending Load: Two small planes located 240 mm apart at the fracture ends were set with fixed constraints. To ensure that the four screws implanted in the proximal tibia were within the span of tibial support, a point was selected on the intersecting line between the middle plane of the two fixed constraints and the implanted steel plate. A 400 N load was applied perpendicularly to the LCP and tibia (Fig. 2c).

Stress distribution cloud maps, fracture end displacement, and stiffness under different loading conditions for each model group were obtained through finite element analysis, and the IFS at the fracture end was calculated. The IFS was defined as the relative change in the fracture gap (∆L) divided by the fracture gap (L) (IFS = ∆L/L), where IFS is the interfragmentary strain, ∆L is the change between the initial length and length under stress, and L is the initial length (10 mm). Owing to the single-model-per-group design, formal statistical hypothesis testing was not performed, and comparisons are presented descriptively on the basis of the calculated values.

## Results

### Stress Distribution of Fixation in Each Model Group under Different Loading Modes

**Fig. 3:**
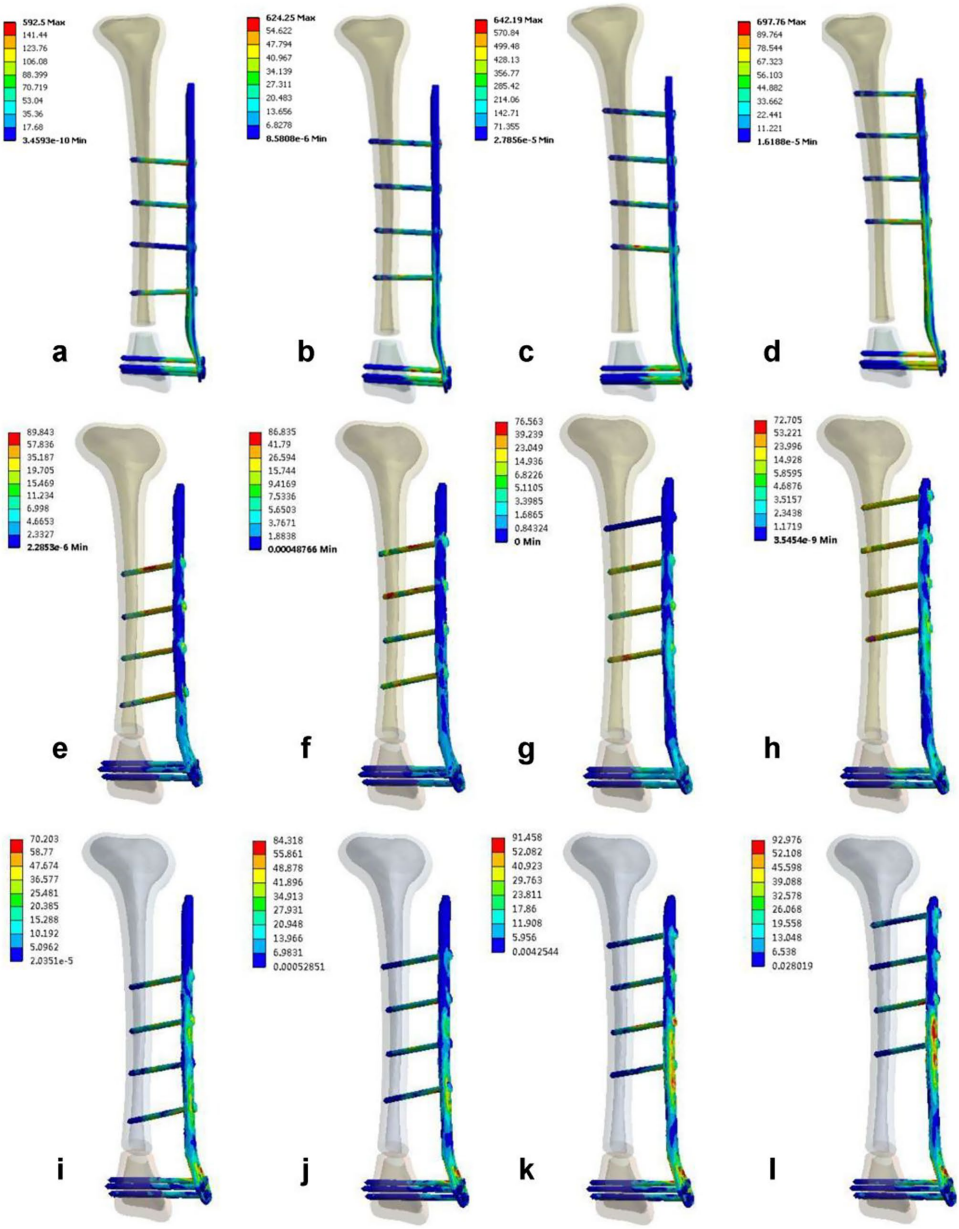
Stress distribution cloud maps of the 4 groups of fixation models under different loading modes. **a**–**d** Axial compression test; **e**–**h** torsion test; **i**–**l** three-point bending test

The maximum stress in the fixation device under axial compression loading for the EF4 group was observed at the L5 connection of the steel plate and the screws (Fig. 3d), with a maximum von Mises stress of 697.96 MPa. Under axial torsion loading, the maximum stress in the fixation device for the EF1 group was observed at the L6 connection of the steel plate and the screws (Fig. 3e), with a maximum von Mises stress of 89.84 MPa. Under a three-point bending load, the maximum stress in the fixation device for the EF4 group was observed at the L5 connection of the steel plate and the screws (Fig. 3l), with a maximum von Mises stress of 92.98 MPa. The stress contour maps for the fixation devices in each group are shown in Fig. 3 (the red arrows represent the location of the maximum von Mises stress), and the specific values of the maximum von Mises stress can be found in Table 3.

**Table 3:** Maximum von Mises stresses of the fixation site and tibia under different loading modes

	Fixation (MPa)			Tibia (MPa)		
	Axial compression	Axial torsion	Three-point bending	Axial compression	Axial torsion	Three-point bending
EF1	592.50	89.84	70.20	268.58	94.26	30.27
EF2	624.25	86.84	84.32	120.76	83.28	33.94
EF3	642.19	76.56	91.46	117.16	81.00	34.33
EF4	697.76	72.71	92.98	235.47	73.09	39.78

### Stress Distribution in the Tibia in Each Model under Different Loading Modes

**Fig. 4:**
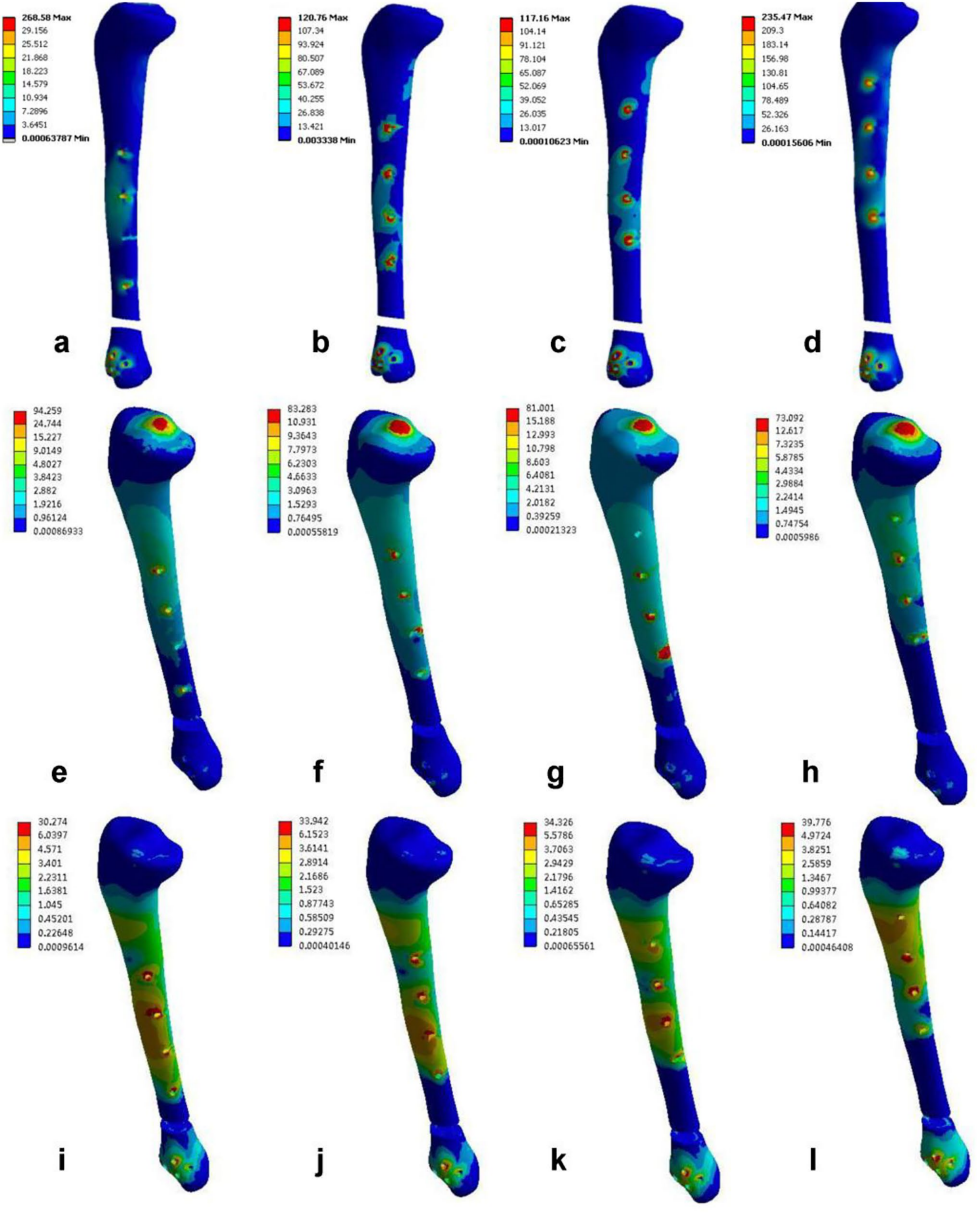
Cloud maps of the tibial stress distribution in the four groups of models under different loading modes. **a**–**d** Axial compression test; **e**–**h** torsion test; **i**–**l** three-point bending test

Under axial compression loading, the tibia stress was greatest in group EF1, which was observed at the proximal most distal foramen ovale of the fracture (Fig. 4a), with a maximum von Mises stress of 268.58 MPa. Under axial torsion loading, the tibia stress was greatest in group EF1, which was observed at the tibial plateau (Fig. 4e), with a maximum von Mises stress of 94.26 MPa. Under three-point bending loading, the tibia stress was greatest in group EF4, which was observed at the proximal most distal foramen orifices of the fracture (Fig. 4l), with a maximum von Mises stress of 39.78 MPa. The stress clouds of the model fixations for each group are shown in Fig. 4 (red arrows represent the site of maximum von Mises stress concentration), and the maximum von Mises stresses are specified in Table 3.

### Fracture End Displacement and Interfragmentary Strain in Each Model Group under Different Loading Modes

Under the three loading modes, the fracture end displacement of the four model groups increased with increasing working length of the steel plate, and the EF1 group had the smallest displacement, followed by the EF2, EF3, and EF4 groups. Under axial compression loading, the fracture end displacements of the four model groups were 7.642 mm, 10.657 mm, 11.003 mm, and 12.219 mm, respectively, with a fracture gap of 10 mm. The interfragmentary strains of the four model groups increased in the following order: EF1 group (76%) < EF2 group (107%) < EF3 group (110%) < EF4 group (122%). The fracture end displacements and interfragmentary strains of the four model groups are detailed in Table 4.

**Table 4:** Comparison of the displacement and interfragmentary strain among the four model groups

	Fracture end displacement (mm)			IFS = ∆L/L
	Axial compression	Axial torsion	Three-point bending	Axial compression (%)
EF1	7.642	0.013	1.270	76
EF2	10.657	0.016	1.869	107
EF3	11.003	0.019	1.903	110
EF4	12.219	0.020	1.949	122

### Stiffness of Fixation in Each Model Group under Different Loading Modes

Under axial compression, axial torsion, and three-point bending loads, the stiffness of the 4 groups of finite element models decreased with increasing working length of the steel plate; the stiffness of the EF1 group was the greatest, followed by those of the EF2, EF3, and EF4 groups. The axial compression, axial torsion, and three-point bending stiffnesses of the EF1 group were 78.50 N/mm, 0.54 Nm/°, and 314.94 N/mm, respectively. Compared with those in the EF1 group, the axial compression, axial torsion, and three-point bending stiffnesses in the EF2 group decreased by 28%, 19%, and 32%, respectively, whereas those in the EF4 group decreased by 37%, 35%, and 35%, respectively. The stiffness of each model group is detailed in Table 5.

**Table 5:** Comparison of implant stiffness under different loading modes

	Axial compression (N/mm)	Axial torsion (Nm/°)	Three-point bending (N/mm)
EF1	78.50	0.54	314.94
EF2	56.30	0.44	214.16
EF3	54.53	0.36	210.22
EF4	49.10	0.35	205.23

## Discussion

Currently, there are conflicting reports on the influence of working length on the stability of LCP structures, with different studies suggesting that increasing the working length of an LCP may be associated with an increase, decrease, or no change in structural stiffness during axial compression testing [25, 26]. Stoffel et al. [26] reported that working length primarily affects axial stiffness and torsional stability and that increasing the distance between the bone and the steel plate leads to decreased stability. To the best of our knowledge, our study is the first to use finite element analysis to investigate the effect of working length on the structural stiffness of femoral LCP fixation for distal tibia fractures. Our study indicated that in all the models, the structural stability of femoral LCP fixation decreased with increasing working length, which is consistent with the findings of Stoffel et al.

Kerkhoffs et al. first described the use of standard AO steel plates as external fixators for the treatment of open fractures and infected nonunions [27]. In recent years, the external application of steel plates has shown satisfactory clinical outcomes, particularly for tibia fractures with weak soft tissue coverage [9–19]. Wu et al. [28] reported a bone healing time of 23.0 ± 5.5 weeks for LCP external fixator patients, which was shorter than that of patients treated with standard external fixation (28.3 ± 6.6 weeks). Zhang et al. [13] evaluated the efficacy of initial locking plate fixation in 28 patients with tibial fractures; the average fracture healing times for AO/OTA classifications 43-A1, 43-A2, and 43-A3 were 14.6 ± 2.67, 17.5 ± 3.66, and 18.4 ± 3.37 weeks, respectively. Luo et al. [10] conducted a systematic review of 12 studies and reported only 2 nonunion cases among 254 patients, resulting in a bone healing rate of 99.2% (95.7%–100%). They reported that the use of locking steel plate fixation achieved satisfactory functional outcomes, high healing rates, and a low incidence of complications.

In recent years, scholars have proposed the concept of biological osteosynthesis (BO) for fractures. BO involves indirect reduction, elastic fixation, preservation of soft tissue and periosteal blood supply, and relative stability to promote callus formation [5]. The stability of the LCP, when used as an internal fixator, depends on the friction between the steel plate and the bone [10]. The LCP external fixation principle is comparable to that of an external fixator, as it provides angular stability [9, 10, 20]. In 2010, Bottlang et al. introduced an improved internal fixation plate technique known as “far cortical locking” [29]. This technique achieves elastic fixation through cantilever bending of the screw shafts for far cortical locking. Its mechanism is similar to that of an external fixator, in which the fixation pins are bent to provide elasticity. By increasing the distance between the bone and the plate, the LCP reduces the stiffness of the internal fixation. This allows for axial micromotion at the fracture site, which stimulates more callus formation for secondary fracture healing. Additionally, this increased distance prevents asymmetrical callus formation caused by the high stiffness of LCP internal fixation and provides a better biomechanical environment for fracture healing [5, 10, 30, 31].

Therefore, the LCP external fixator is an exceptional treatment option for fractures that offers numerous advantages [10, 19, 24]. Research has demonstrated that steel plates are capable of shielding soft tissues and supplying blood near fracture sites, thus reducing the necessity for early open reduction surgery. The thick plate, along with multiple screw holes and a long locking screw diameter, provides exceptional stability by securing both sides of the bone cortex. The low profile of the LCP makes it possible for patients to conceal it with regular clothing, and angular stability prevents ankle joint fixation by allowing early functional exercise. Moreover, the external fixator can be easily removed without anesthesia, which minimizes secondary surgical trauma. Nonetheless, LCP external fixation has several drawbacks, such as the need for anatomical reduction before fixation and the potential difficulties in operating and adjusting the locking plate. Overall, the LCP external fixator is a highly effective and secure form of treatment for fractures, with several benefits and minimal risks for patients.

However, only a few studies have investigated the biomechanical aspects of LCP external fixation, and the results of these studies have been inconsistent [9, 18–24]. Kanchanomai and Phiphobmongkol [23] evaluated the effects of different fracture gap sizes (1, 5, and 10 mm) on the stability of LCP external fixation for tibial fractures (with a bone‒plate distance of 30 mm). The results revealed that the stiffness of the 1 mm fracture gap (stable tibial fracture) was similar to that of an intact tibia, whereas the stiffnesses of the 5 mm and 10 mm fracture gaps were significantly lower than those of an intact tibia and the 1 mm fracture gap. Liu et al. [24] reported that the compressive stiffness of femoral LCP external fixation and the torsional stiffness of tibial LCP external fixation were significantly lower than those of tibial LCP internal fixation. However, the torsional stiffness of femoral LCP external fixation was significantly greater than that of tibial LCP internal fixation, and both the compressive and torsional stiffnesses of femoral LCP external fixation were superior to those of tibial LCP external fixation. Therefore, they concluded that the femur was more suitable for LCP external fixation and that partial weight-bearing could be allowed for stable fracture patients, but caution should be exercised in the early stages of treatment for unstable tibial shaft fractures. Makelov [9] and colleagues used finite element analysis to establish the stability of a less invasive stabilization system for distal femur internal fixation (bone‒plate distance of 2 mm) and external fixation (bone‒plate distances of 22 mm and 32 mm) for unstable tibial fractures (fracture gap of 20 mm). Their results indicated that caution regarding patient activity should be exercised in the early postoperative period, and partial weight bearing could be allowed when tibial fractures with a bone‒plate distance of 30 mm are fixed. Therefore, in this study, a 10 mm fracture gap was created 50 mm above the ankle joint to represent unstable fractures of the distal tibia.

Two studies have explored the effects of bone plate spacing on LCP stability in distal tibia fractures treated with LCP external fixation. The first study, conducted by Ma et al. [21], utilized finite element analysis to investigate the impact of bone plate spacing on axial and torsional stiffness. The study revealed that increases in bone plate spacing of 6 cm and 10 cm resulted in a significant reduction in axial stiffness of 84% and 94%, respectively, and a reduction in torsional stiffness of 12%–21% in comparison with the LCP internal fixation model. The authors concluded that while LCP external fixation decreases axial and torsional stiffness, a reduction in structural strength may promote callus formation at the fracture site, thereby facilitating fracture healing. The second study, conducted by Zhang et al. [22], also employed finite element analysis to investigate the effect of the bone plate distance on LCP stability in distal tibia fractures with LCP external fixation of the femur. The study revealed a greater increase in stiffness in the 1-mm, 10-mm, and 20-mm groups than in the other groups, suggesting possible stress shielding. The authors recommended that the distance between the plate and bone should be less than 30 mm to ensure the stability of distal tibia fractures, which may be beneficial for inducing callus tissue formation. In the clinical application of external fixation, the distance between the external fixator and the bone depends on the degree of soft tissue swelling and the thickness of the soft tissue. Therefore, individual differences in skin and soft tissue thickness affect the bone plate distance, which subsequently depends on the degree of soft tissue swelling and soft tissue thickness of the patient’s medial calf. In our study, a bone plate distance of 30 mm was chosen on the basis of clinical practice.

The investigations cited above examined the effects of certain variables, such as fracture gap size and plate distance, on the stability of femoral LCP external fixation. Notably, the influence of the working length of the plate, specifically the screw distribution, was not assessed in these studies. Research has demonstrated that the exclusion of a screw hole in proximity to the fracture site can severely reduce axial and torsional stiffness. In fact, each additional unoccupied screw hole may decrease the stability by approximately 10% [26]. The results of this study revealed that the omission of one screw near the proximal end of the fracture yielded axial and torsional stiffnesses of 78.50 N/mm and 0.54 Nm/°, respectively, for the EF1 group. Compared with the EF1 group, the EF2 group (omitting 2 screws) exhibited significant reductions in axial and torsional stiffness of 28% and 19%, respectively. Similarly, the EF4 group (omitting 4 screws) demonstrated significant reductions of 37% and 35%, respectively. While the fixator structures, loading patterns, and results of the present study differ from those of the referenced studies, they imply that the working length has a discernible effect on the compressive and torsional stiffness of the LCP external fixation. Plate length and screw density have also been identified as factors influencing LCP stability and callus formation at fracture sites. Longer plates are associated with greater stability, and a lower screw density in proximity to the fracture site promotes callus growth [2, 10, 25, 31]. Thus, for this study, a longer femoral LCP (18 holes) with sparsely distributed fixation screws was selected for modeling. A longer plate increased the stability of the LCP, whereas a reduced screw density favored callus formation at the fracture site.

It has been suggested that an appropriate LCP working length should be used for fixation of the fracture end, and working lengths that are too short or too long are likely to result in stress concentration in the plate or insufficient stability, which often leads to failure of fracture fixation and malunion, etc. [10, 31–33]. In the study of Stoffel et al., one sample had fixation failure when 4 screws were omitted from the fracture end of the plate. The authors concluded that for stable fractures (1 mm fracture gap), the fracture end contact can be fixed with 1–2 screws omitted from both ends of the fracture, whereas for comminuted fractures, the screws should be fixed as close to the fracture end as possible and that excessive working lengths tend to lead to insufficient plate stability [26]. In our study, when the EF4 group was fixed with 4 screws omitted from the proximal end of the fracture, the stress value of the tibia under axial compression loading (235.47 MPa) was similar to that of the EF1 (268.58 MPa) group. This value was significantly greater than those of the EF2 (120.76 MPa) and EF3 (117.16 MPa) groups, which we considered to be caused by the insufficient stability of plate fixation at the fracture end.

Research has shown that the length of the plate used (screw distribution) and the type of screws employed significantly impact the strain in the fracture gap. The IFS determines the type of fracture healing, which in turn affects the healing of the fracture [33–36]. Mardian et al. [34] used finite element methods to investigate the effect of the working length of a locking plate on the interfragmentary motion of distal femoral fractures under physiological loads. The working length, as determined by the screw position, significantly affects IFS and therefore has an impact on fracture healing. According to Claes [35], when the IFS is less than approximately 5%, intramembranous ossification occurs. When the IFS reaches approximately 15%, endochondral ossification occurs. When the IFS is between 15% and 30%, mainly fibrous tissue and fibrocartilage are formed. This approach can harden the cartilage and reduce the IFS to less than 15%, allowing bone healing through endochondral ossification. Therefore, from a biomechanical perspective, the ideal internal fixation device should achieve approximately 10% to 30% IFS in the fracture gap. An IFS below 5% or above 30% is not conducive to stimulating callus formation, which can lead to delayed healing or nonunion of the fracture. Research has shown that for fracture gaps of 0.7 to 3 mm, an IFS of 0.2 mm to 1 mm, which corresponds to 13% to 33% of the IFS, can stimulate the formation of good callus tissue [35]. Makelov et al. [9] conducted a virtual biomechanical analysis that demonstrated that under partial weight-bearing conditions (250 N), the IFS remains within the range required for normal phase II fracture healing. The EF1 group in this study included a patient weighing 60 kg under full weight-bearing conditions (600 N), and the IFS (76%) was far above the ideal range (10%–30%). Therefore, on the basis of the results of this study, it is recommended that patients undergoing distal tibial fracture treatment via this fixation method be partially weight-bearing in the early stages. Full weight-bearing should be carefully considered.

In our previous study [19], biomechanical tests were performed to study the stability of distal tibia fractures using femoral LCP external fixation (with a plate spacing of 30 mm), and the results were compared with those of tibial LCP internal fixation, tibial LCP external fixation and conventional external fixation. Under axial compression and torsional loading, the stiffnesses of the femoral LCP were 78.42 N/mm and 0.48 Nm/°, whereas those of the tibial LCP external fixation were 34.03 N/mm and 0.24 Nm/°, respectively. The study concluded that femoral LCP external fixation was superior to tibial LCP external fixation, which is in agreement with the findings of Liu et al. [24]. Hoenig et al. reported that the mean compressive stiffness was 72.5 Nm/mm for standard plates, 122 Nm/mm for LCP nails and 179 Nm/mm for intramedullary nails [37]. It has been shown that the Ilizarov fixator has a stiffness between 73 and 79 N/mm [24]. The finite element analysis results of this study revealed that the stiffnesses of the EF1 group under axial compression and torsion were 78.50 N/mm and 0.54 Nm/°, respectively, which was consistent with our previous biomechanical test results; thus, we considered the model valid. Despite the different fixator configurations and loading patterns used in the above studies, the results are still useful for assessing the stiffness of the femoral LCP as an external fixator. On the basis of these results, we found that the stiffness of the femoral LCP as an external fixator was similar to that of a standard plate or Ilizarov fixator and superior to that of tibial LCP external fixation. Therefore, this study suggests that femoral LCP external fixation for lower tibial fractures can be considered a temporary or definitive fixation method.

Limitations of this study must be considered: 1. The effects of fibular and muscle forces were not considered, and this experiment was a static test that could not simulate the stability of the plate during motion. 2. Further fatigue testing should be performed on LCP externally fixed lower tibia fracture models to determine the effect of locking screw contact settings on the dynamic stability of each model. 3. Although the four screw distribution patterns in this study are representative of those used in clinical practice, they do not encompass all clinical possibilities; common configurations such as asymmetric screw placement, variation in the number of screws, variation in screw angles, or the use of plates of different lengths were not considered. Our team will continue to improve subsequent tests to provide clinicians with a sufficient biomechanical basis for the use of femoral LCP external fixation in the treatment of lower tibia fractures.

## Conclusion

Finite element analysis revealed that the structural stiffness of the femoral LCP external fixator decreased with increasing working length (determined by the screw position) under full weightbearing, which led to an increase in IFS at the fracture end; additionally, the structural stiffness (78.50 N/mm in compression and 0.54 Nm/° in rotation) and IFS values were outside the ideal range required for second-stage healing. Therefore, for unstable fractures of the lower tibia, femoral LCP external fixation screws should be placed as close to the fracture end as possible to increase stability and facilitate fracture healing.

## Data Availability

The datasets used and/or analyzed during the current study are available from the corresponding author upon reasonable request.
